# Psychology looking forward

**DOI:** 10.1038/s44271-023-00004-1

**Published:** 2023-07-25

**Keywords:** Human behaviour

## Abstract

The aim of Communications Psychology is to promote open research across all areas of psychology. We are looking forward to working with researchers to publish high-quality research; to offer the community an accessible platform for review and debate; and to shape our practices to serve a field embracing open science.

**Figure:**
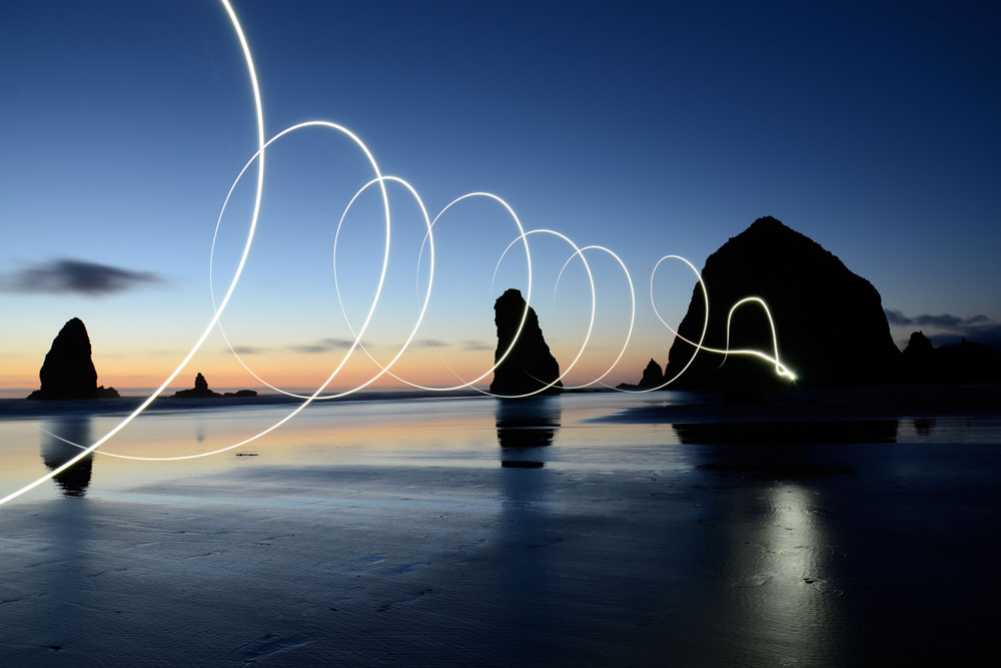
Photo by Jamison McAndie on Unsplash

No topic that currrently dominates public debate, be it AI, mitigating climate change, or achieving just and equitable societies, is unrelated to questions that are pursued in psychological research. Clearly, psychology on its own does not offer the solutions. Yet, the questions that psychology seeks to answer—how humans perceive, judge, relate, and respond to their social and physical environment—are fundamentally a part of addressing these interdisciplinary challenges. At *Communications Psychology*, we believe that all areas of psychology play a part in informing progress to this end. And that psychology as a field is engaged in a productive process of overcoming its own challenges allowing it to make precisely that contribution.

*Communications Psychology* is a community-focused journal which publishes high-quality, rigorous work of interest to specialists working in any area of psychology and neighboring disciplines. Research at the journal is handled collaboratively by a team of professional in-house editors and academics who serve as editorial board members. We all subscribe to and maintain the same editorial bar and policies. The journal is strongly committed to the values of open research, and these values inform the practices we put into place to respond to researchers’ needs.

The journal places an emphasis on the values of transparency and accountability. We hope that the policies that flow from these norms will earn the trust of the community that will allow the journal to make a positive contribution to the discipline. This includes the trust of our readers that we publish carefully vetted high-quality research; the trust of our authors that their research will be treated fairly regardless of who they are and in which area of psychology they work; and the trust of our referees that we respect their advice. We therefore make transparent the decision process that led to the publication of each individual article. Peer-reviewed publications are accompanied by a file containing the editorial decision letters and referee reports. Diving into the transparent peer-review file of our first published Article^1^ will tell readers that the editors were initially reluctant to invite a revision of the paper, given the amount of work that would be required of the authors to address the referees’ concerns. This decision follows from our belief that it is unfair to authors to ask for excessive additional work when the outcome is unclear. However, readers will also find that the authors were able to respond to the referees’ concerns based on the data they had, leading to a positive outcome.

When we opened for submissions  in October 2022 we also launched our first Collection, focused on Replication and Generalization. Although the call for papers to be included in the Collection will close this year, the commitment to publishing replication and generalization work is not a passing interest: it lies at the heart of the journal’s aims.

We explicitly welcome submissions of replication and generalization projects in the Registered Report format, which we believe fosters a more constructive review culture. We equally welcome Registered Reports that address new research questions, and we were delighted that the first manuscript to receive acceptance in principle at the journal was a Stage 1 Registered Report protocol which is publicly deposited on our figshare repository^2^.

In addition to standard Research Articles and Registered Reports, the journal also publishes high utility data and code in the Resource format. Data and code are as much products of research as the insights they offer into specific questions. Publishing data and code with high reuse value means that other researchers can benefit from the authors’ efforts, while the authors receive credit for their contribution.

Universal access to scientific knowledge is a widely shared principle of the open research movement. All publications in *Communications Psychology* are published Open Access (under a CC-BY license). We understand that opening research is not limited to providing greater access to its insights, but also about widening participation in research, with researchers from across the world publishing science on humans from across the globe.

Researchers from low and middle income countries may find information about APC waivers helpful and authors from other locations who face financial need are encouraged to contact the journal prior to submission.

Journals can and must play a role in leveling the playing field. We strive to work with a gender-balanced, diverse editorial board, and constantly work to expand and diversify our reviewer pool. We do not disclose author information in our reviewer invitation letters and we offer double-anonymized peer review at the authors’ discretion. We encourage authors to consider this route not only if they fear their identity puts them at a disadvantage, but also if they believe in the value of helping reviewers to focus on the work rather than on its origin.

Our review and opinion content presents new ideas, theory development, and overview articles on specific research topics in psychological research. For example, the Perspective by Lavan & McGettigan^3^ presents a new model for person perception from voices that explains both familiar and unfamiliar voice processing. However, we also explicitly commission and invite content that provides a platform for the concerns of researchers and themes that are historically or presently marginalized in the psychological research community.

The Perspective by Korbmacher and colleagues from the FORRT project^4^ emphasizes how the open research revolution in psychology has come a long way. We agree that it is time to look forward to what the field can achieve.

The editors at *Communications Psychology* want to work with and for the psychology community. We value and look forward to your contributions and input.
